# Expression, regulation and clinical significance of soluble and membrane CD14 receptors in pediatric inflammatory lung diseases

**DOI:** 10.1186/1465-9921-11-32

**Published:** 2010-03-19

**Authors:** Veronica Marcos, Phillip Latzin, Andreas Hector, Sebastian Sonanini, Florian Hoffmann, Martin Lacher, Barbara Koller, Philip Bufler, Thomas Nicolai, Dominik Hartl, Matthias Griese

**Affiliations:** 1 Children's Hospital of the Ludwig-Maximilians-University, Munich, Germany; 2 Children's Hospital, University of Berne, Switzerland; 3 Department of Dermatology and Allergy, Ludwig-Maximilians-University, Munich, Germany; 4 Department of Surgery, Klinikum Harlaching, Munich, Germany

## Abstract

**Background:**

Inflammatory lung diseases are a major morbidity factor in children. Therefore, novel strategies for early detection of inflammatory lung diseases are of high interest. Bacterial lipopolysaccharide (LPS) is recognized via Toll-like receptors and CD14. CD14 exists as a soluble (sCD14) and membrane-associated (mCD14) protein, present on the surface of leukocytes. Previous studies suggest sCD14 as potential marker for inflammatory diseases, but their potential role in pediatric lung diseases remained elusive. Therefore, we examined the expression, regulation and significance of sCD14 and mCD14 in pediatric lung diseases.

**Methods:**

sCD14 levels were quantified in serum and bronchoalveolar lavage fluid (BALF) of children with infective (pneumonia, cystic fibrosis, CF) and non-infective (asthma) inflammatory lung diseases and healthy control subjects by ELISA. Membrane CD14 expression levels on monocytes in peripheral blood and on alveolar macrophages in BALF were quantified by flow cytometry. *In vitro *studies were performed to investigate which factors regulate sCD14 release and mCD14 expression.

**Results:**

sCD14 serum levels were specifically increased in serum of children with pneumonia compared to CF, asthma and control subjects. *In vitro*, CpG induced the release of sCD14 levels in a protease-independent manner, whereas LPS-mediated mCD14 shedding was prevented by serine protease inhibition.

**Conclusions:**

This study demonstrates for the first time the expression, regulation and clinical significance of soluble and membrane CD14 receptors in pediatric inflammatory lung diseases and suggests sCD14 as potential marker for pneumonia in children.

## Introduction

Inflammatory lung diseases of infective or non-infective origin are among the leading morbidity and mortality factors in children and require early diagnosis for specific treatment to prevent disease progression and chronic lung remodelling [1,2]. Therefore, novel strategies for early detection of inflammatory and infective lung diseases in childhood are of high interest.

Lipopolysaccharide (LPS) is recognized by the human immune system via binding to LPS binding protein (LBP) and transferrring the LPS/LBP complex to CD14 [3,4]. CD14 is a myeloid differentiation antigen that is mainly produced by monocytes and macrophages. CD14 acts as a receptor for bacterial LPS in cooperation with Toll-like receptor 4 (TLR4) [5]. The binding of LPS via LBP and CD14 to TLR enhances mitogen activated protein kinase (MAPK) signalling and promotes the secretion of pro-inflammatory cytokines and chemokines [6]. CD14 can bind bacterial ligands and receptors on phagocytes, thereby mediating phagocytosis of bacteria and clearance of apoptotic cells [3,7,8].

CD14 exists as a soluble (48/56 kDa) and membrane-associated glycosylphosphatidylinositol (GPI)-anchored (55 kDa) protein, present on the surface of monocytes, macrophages, dendritic cells and neutrophils [4,8]. The soluble form of CD14 (sCD14) is produced either by proteolytic cleavage or by secretion without the GPI moiety by monocytes [9,10]. Soluble CD14 is detectable both in serum and bronchoalveolar lavage fluid (BALF) [11]. Recently, Dressing et al. demonstrated in a murine model that *Streptococcus pneumoniae *utilizes sCD14 in the bronchoalveolar space to cause invasive respiratory tract infections [12]. When viewed in combination, sCD14 is deemed to act as a key component in pulmonary inflammation/infection and may represent a promising marker and therapeutic target in respiratory diseases.

The expression, regulation and clinical significance of sCD14 and mCD14 in pediatric lung diseases has not been defined. Therefore, we quantified sCD14 and mCD14 levels in peripheral blood and BALF of children with infectious and non-infectious pediatric lung disease and healthy control groups. Furthermore, we examined which factors induce the release of sCD14 by peripheral blood mononuclear cells (PBMCs) *in vitro*.

## Methods

### Study design

Soluble and membrane CD14 expression levels were analyzed in serum and BALF of age-matched children with pneumonia (n = 48 all pneumonia, n = 31 bacterial pneumonia), cystic fibrosis (CF, n = 39); allergic asthma (n = 15) and healthy control subjects (n = 8) (table 1). The pneumonia group included 48 children with a mean age of 11 ± 4 (SD) years. All pneumonia patients were inpatients of the Children's hospital of the University of Munich and underwent detailed diagnostic work-up. BAL was initiated by the attending physician for further diagnostic clarification, in particular since a large proportion of the included patients had chronic pulmonary symptoms. Pneumonia patients were stratified in 'bacterial pneumonia' and 'non-bacterial pneumonia'. Bacterial pneumonia was diagnosed when the following criteria were given (i) infiltrates in chest radiographs, (ii) increased C-reactive protein (CRP), elevated white blood cell count (WBC) and/or accelerated erythrocyte sedimentation rate (ESR), (iii) clinical signs of pneumonia (cough, dyspnoe, tachypnoe, fever) and (iv) detection of bacterial pathogens in BALF. The bacterial pneumonia group included 19 male and 12 female children. The CF group included 23 male and 16 female patients with a mean age of 11 ± 6 (SD) years. Inclusion criteria were the diagnosis of CF by clinical symptoms and positive sweat tests or disease-inducing mutations, forced expiratory volume in 1 second (FEV_1_) > 25% of predicted value and being on stable concomitant therapy at least 2 weeks prior to the study. Among all 39 CF patients, 17 patients were colonized with *P. aeruginosa *and 19 patients with *S. aureus*. Twenty-five CF patients were ΔF508 homozygous, nine were ΔF508 heterozygous carriers of the CFTR gene and five had other CFTR mutations than ΔF508. The CF patients had moderate to severe disease severity, as defined by the activity and physical examination criteria of the scoring system of Shwachman and Kulczycki [13]. All CF patients were clinically stable at least 2 months prior to the study, as indicated by lack of self-reported change in symptoms over the preceding 2 months, and none reported a change in airway symptoms in the 2 months prior to the study. BAL was performed for further diagnostic clarification. The asthma group included 7 male and 8 female patients with a mean age of 10 ± 4 (SD) years. Inclusion criteria were recurrent episodes of wheezing and objective evidence of asthma as indicated by β2-agonist-reversible airflow obstruction (≥12% improvement in FEV1 % predicted), bronchial hyperresponsiveness (exercise challenge) and ≥20% intraday peak flow variability, positive skin prick testing (wheal diameter of ≥3 mm to at least one common allergen), elevated total serum IgE (>150 kU/ml; IgE-Elecsys, Roche, Basel, Switzerland), and/or the presence of specific IgE (RAST class >2). The RAST was performed for forty inhalation and food allergens (Sanofi Diagnostics Pasteur, Inc, Chaska, MN). All asthma patients used inhaled bronchodilators and nine asthma patients used inhaled corticosteroids. Spirometry and flow volume curves were performed according to the ATS guidelines [14]. The clinical indication for BAL in pediatric asthmatic patients was the severity and chronicity of the asthmatic disease that prompted us to perform BAL for a more extensive diagnostic workup, in order to exclude non-asthmatic causes for the chronic pulmonary disease. Eight age-matched control subjects without pulmonary diseases were selected as the control group (5 male, 3 female; mean age: 9 ± SD 4 years) as described previously [15,16]. These subjects had no suspected or proven pulmonary disease and were free of respiratory tract infections. The control subjects underwent minor surgical interventions and BAL was performed prior to the surgical procedure for research purposes. This study was approved by the institutional review board of the LMU Children's hospital. Written informed consent was received from all patients or their parents.

**Table 1:** Patient groups

	Pneumonia		Cystic fibrosis	Asthma	Controls
				
	bacterial	non-bacterial			
N	31	17	39	15	8
Age [yrs]	10 ± 5	12 ± 7	11 ± 6	10 ± 4	9 ± 4
Sex (m:f)	19/12	9/8	23/16	7/8	5/3
CRP (mg/l)	114 ± 64	53 ± 25	8 ± 3	12 ± 4	2 ± 1
WBC (10^9^/l)	20 ± 8	11 ± 5	9 ± 4	10 ± 5	8 ± 2
Atopy	4/31	5/17	11/39	15/15	0/8
FEV_1 _(% pred)	-	-	95 ± 20	68 ± 12	-
FVC (% pred)	-	-	93 ± 19	73 ± 26	-
Bacteria detected	31	0	30/39	3/15	0/8

BALF cells					
Viability (%)	76 ± 20	70 ± 14	76 ± 34	81 ± 26	85 ± 13
Recovery (%)	62 ± 24	52 ± 18	42 ± 17	56 ± 11	58 ± 18
Total cells (10^3^/ml)	962 ± 303	712 ± 144	3240 ± 9380	240 ± 113	145 ± 23
Neutrophils (%)	38 ± 24	10 ± 13	63 ± 31	16 ± 23	2 ± 1
Macrophages (%)	55 ± 28	62 ± 34	33 ± 16	74 ± 21	87 ± 9
Lymphocytes (%)	12 ± 16	20 ± 11	5 ± 3	13 ± 14	5 ± 3
Eosinophils (%)	4 ± 2	2 ± 3	2 ± 1	5 ± 2	0 ± 0
Mast cells (%)	1 ± 1	1 ± 2	1 ± 1	2 ± 1	0 ± 1
Plasma cells (%)	4 ± 7	8 ± 5	3 ± 4	6 ± 4	2 ± 1
BALF cells analyzed by flow cytometry	15/31	8/17	9/39	13/15	8/8

results are expressed as means ± SD; m: male, f: female; WBC: white blood count; FEV_1_: Forced expiratory volume in 1 second (% of predicted); FVC: Forced vital capacity (% of predicted); BALF: bronchoalveolar lavage fluid; CRP: C-reactive protein; WBC: white blood cell count; *Pathogens detected in any patients sample at time of study (blood, BALF or sputum).

### Bronchial alveolar lavage

Bronchoscopy and BAL (4 × 1 ml of 0.9% NaCl per kg body weight) were performed as described previously [17]. The obtained BALF was filtered through two layers of sterile gauze and was centrifuged at 200 g for 10 minutes. The cell pellet immediately underwent flow cytometric analysis of mCD14 expression on alveolar macrophages as described below.

BALF was divided into two samples: one for cytospin preparation and sCD14 analysis and one for quantitative bacterial culture. Cytospins were performed out of native BALF. Differential cell counts were obtained from cytospins stained with May-Gruenwald-Giemsa (Diff-Quik; Baxter Diagnostic AG, Düdingen, Switzerland). At least 600 cells were counted in each subject. Pathogens in BALF were detected as described previously [18].

### ELISA

sCD14 levels were measured in duplicates by a commercially available, sandwich enzyme-linked immunosorbent assay (ELISA) kit (Immunobiological Laboratories, Hamburg, Germany) according to the manufacturer's instructions. We performed initial studies to test whether processing of CF BAL affects sCD14 quantification. These studies showed that filtration and centrifugation of CF BAL had no significant effect on sCD14 levels. The lower detection limit of the assay was 7 ng/ml. Intra-assay variability was determined by evaluating 5 serum samples 10 times within the same assay run and showed a coefficient of variation (CV) between 5% and 8%. Inter-assay variability was determined by measuring 5 serum samples in 5 consecutive assay runs and showed a CV between 6% and 11%.

### Flow cytometry

The following monoclonal anti-human antibodies were used (all from BD Pharmingen, San Diego, CA, USA): mouse IgM CD15 FITC, mouse IgG_1 _CD45 APC, mouse IgG_2a _CD14-Cy5.5, mouse IgG_2b _CD68-PE, mouse IgM FITC, mouse IgG_1 _APC, mouse IgG_2a _Cy5.5 and mouse IgG_2b _PE. Peripheral blood cells and BALF cells were analyzed by flow cytometry. Monocytes in peripheral blood were detected according to their forward-/side-scatter characteristics (FSC/SCC). Alveolar macrophages in BALF were detected as described previously [19,20]. Alveolar macrophages were detected by positive expression for CD45, negative staining for CD15 and their high expression of CD68. For antibody staining, cells were incubated with the respective antibodies for 40 minutes and analyzed by flow cytometry (FACSCalibur, Becton-Dickinson, Heidelberg, Germany). Isotype controls were subtracted from the respective specific antibody expression and the results were reported as mean fluorescence intensity (MFI). Calculations were performed with Cell Quest analysis software (Becton-Dickinson, Heidelberg, Germany).

### *In vitro *stimulation

PBMCs were isolated from human peripheral blood by Ficoll-Hypaque (Amersham Pharmacia, Piscataway, NJ) gradient centrifugation. Recombinant flagellin (*Salmonella typhimurium*, TLR5 ligand), nonmethylated CpG-oligonucleotides type A (5'ggGGGACGATCGTCgggggg 3', stimulatory TLR9 ligand), peptidoglycan (PGN; from *Staphylococcus aureus*, TLR2/NOD2 ligand), the synthetic bacterial lipoprotein Pam_3_CysSerLys_4 _(Pam_3_CSK_4_, TLR1/2 ligand), zymosan A *(Saccharomyces cerevisiae*, TLR2/6 ligand) and LPS from *E. coli *(TLR4 ligand)were from Invivogen (San Diego, CA, USA). R848 (resiquimod hydrochloride; a single-stranded RNA analog; TLR7/8) was purchased from GL Synthesis (Worcester, MA, USA). Polyinosine-polycytidylic acid (poly I:C) (a double-stranded RNA analog; TLR3) was obtained from Pharmacia (Uppsala, Sweden). Phorbol myristate acetate (PMA) and phenylmethylsulfonyl fluoride (PMSF) were from Sigma-Aldrich (St. Louis, MO, USA). All reagents, buffers and media were free of LPS (<0.01 ng/ml) by Limulus assay (Sigma-Aldrich).

Since it has been reported that LPS modulates sCD14 and mCD14 most significantly upon long-term incubation (>36 hours)[21], PBMCs (2 × 10^6^) were stimulated in RPMI medium for 1 hour or 40 hours with PMA (10 ng/ml), fMLP (100 ng/ml) and of LPS (100 ng/ml), Pam_3_CSK_4 _(1 μg/ml), PGN (1 μg/ml), poly I:C (50 μg/ml), R848 (10 μg/ml), CpG-DNA (100 μg/ml), flagellin (1 μg/ml) or zymosan (50 μg/ml) at 37°C. Where indicated, PBMCs (2 × 10^6^) were pretreated for 1 hour with the protease inhibitor PMSF (10 mM) and were then stimulated for 40 hours at 37°C with RPMI, CpG-DNA (100 μg/ml) or LPS (100 ng/ml).

### Statistical analysis

Since the data distribution was non-parametric, results are given as medians with ± interquartile ranges (IQRs) or medians with ranges. Comparisons among all groups were performed with the Kruskall-Wallis test and comparisons between two patient groups were performed with the Mann-Whitney *U *test. Correlation analysis was performed by calculating the two-tailed Spearman rank correlation test [22]. Diagnostic value of the serological markers for diagnosis of bacterial pneumonia and receiver operator characteristics (ROC) curves were calculated using STATA^® ^version 8.2 for Windows (STATA Corporation, College Station, TX, USA). Cut-off levels were set at the level that resulted in the highest diagnostic accuracy, defined as correctly positive classified plus correctly negative classified as percentage of all.

## Results

### Soluble CD14 is increased in pediatric pneumonia

**Figure 1:**
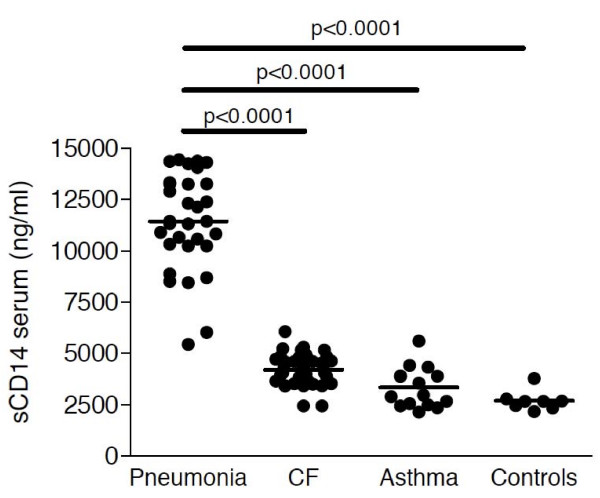
**Soluble CD14 levels in serum**. Soluble CD14 levels were analyzed by ELISA in serum of children with pneumonia (n = 48), CF (n = 39), asthma (n = 15) and control children without pulmonary diseases (n = 8). Horizontal bars represent medians.

**Figure 2:**
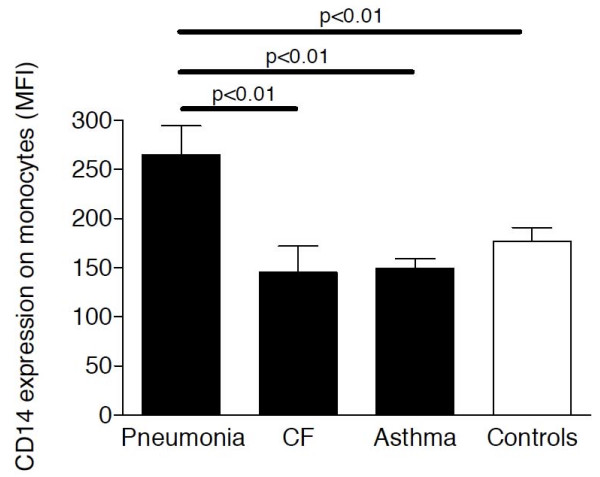
**Membrane CD14 expression in peripheral blood**. Membrane CD14 expression levels were analyzed by flow cytometry in peripheral blood of children with pneumonia (n = 48), CF (n = 39), asthma (n = 15) and control children without pulmonary diseases (n = 8). Bars represent medians ± IQRs. MFI: mean fluorescence intensity.

Soluble CD14 levels were significantly increased in serum of children with pneumonia (including patients with bacterial and non-bacterial pneumonia; median: 11433 ng/ml; range: 5429-15460 ng/ml) compared to CF (median: 4168; ng/ml; range: 2437-6061 ng/ml), asthma (median: 2960; ng/ml; range: 2134-5588 ng/ml) and control (median: 2654; ng/ml; range: 2154-3764 ng/ml) subjects with almost no overlap between pneumonia patients and the other patient groups (Figure 1). Soluble CD14 serum levels of children with CF or asthma did not differ from sCD14 levels of control children. Similar to sCD14 levels, mCD14 expression levels on peripheral blood monocytes were increased in pneumonia patients compared to CF, asthma and control children (Figure 2).

**Figure 3:**
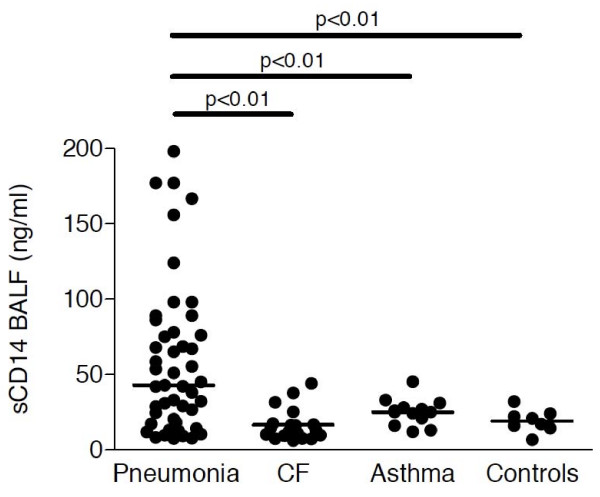
**Soluble CD14 levels in BALF**. Soluble CD14 levels were analyzed by ELISA in BALF of children with pneumonia (n = 48), CF (n = 39), asthma (n = 15) and control children without pulmonary diseases (n = 8). Horizontal bars represent medians.

**Figure 4:**
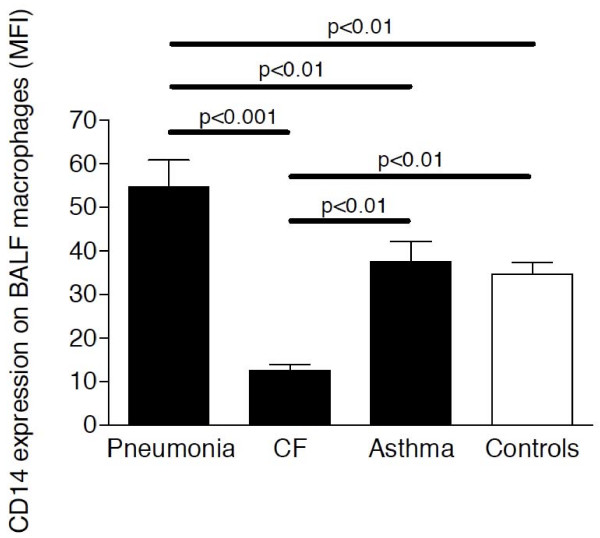
**Membrane CD14 expression in BALF**. Membrane CD14 expression levels were analyzed by flow cytometry in BALF of children with pneumonia (n = 48), CF (n = 39), asthma (n = 15) and control children without pulmonary diseases (n = 8). Bars represent medians ± IQRs. MFI: mean fluorescence intensity.

In BALF, sCD14 levels were also significantly increased in children with pneumonia (median: 43 ng/ml; range: 8-198 ng/ml) compared to CF (median: 13 ng/ml; range: 6-44 ng/ml), asthma (median: 25; pg/ml; range: 12-45 pg/ml) and control (median: 19 pg/ml; range: 7-32 pg/ml) subjects, but overlapped substantially with levels of non-pneumonia patients (Figure 3). Soluble CD14 levels in BALF of children with CF or asthma did not differ from sCD14 levels of control children. Similar to sCD14 levels, mCD14 expression levels on BALF macrophages were increased in pneumonia patients compared to CF, asthma and control children (Figure 4).

**Figure 5:**
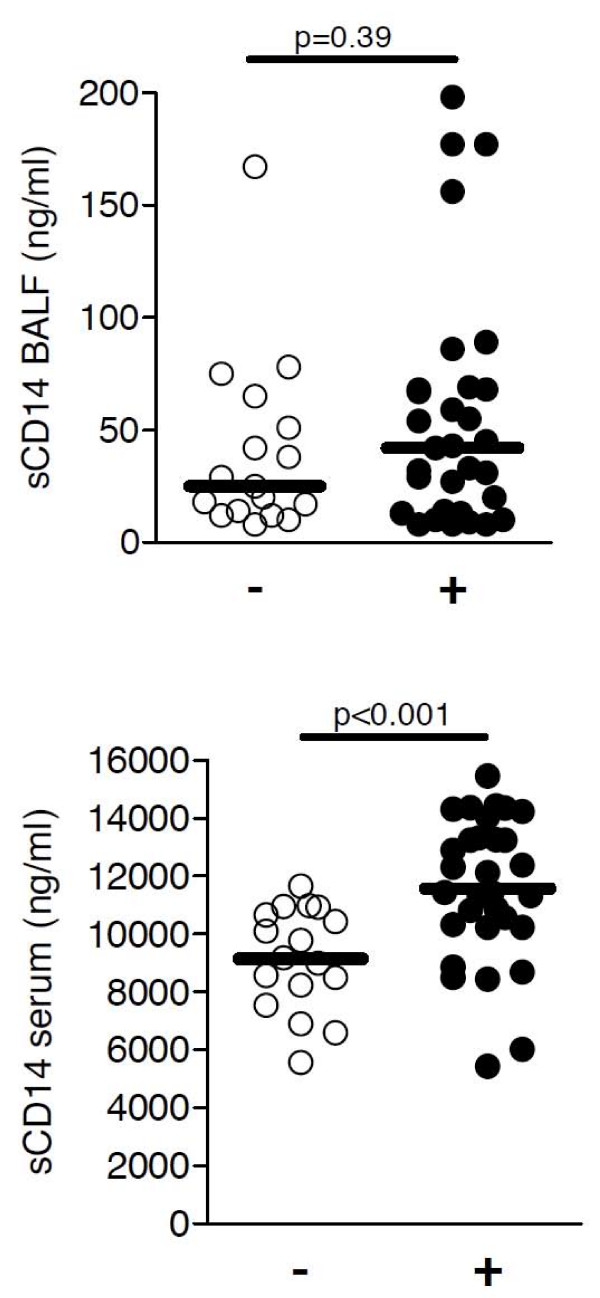
**Soluble CD14 levels and bacterial pneumonia**. BALF (A) and soluble (B) sCD14 levels are shown for pneumonia children with (+, n = 31) or without (-, n = 17) bacterial pathogens detected. Comparisons among all groups were performed with the Kruskall-Wallis test and comparisons between two patient groups were performed with the Mann-Whitney *U *test. Horizontal bars represent medians.

Stratifying pneumonia patients in bacterial and non-bacterial patients, children with bacterial pneumonia had significantly higher levels of sCD14 in serum, but not in BALF, compared to pneumonia patients without detection of bacterial pathogens (Figure 5). Among children with bacterial pneumonia, patients with detection of *Streptococcus pneumoniae *had higher sCD14 levels compared to other patients (data not shown). A positive correlation between sCD14 and mCD14 levels was found in BALF of pneumonia patients (r = 0.47, p < 0.05) and in peripheral blood of pneumonia (r = 0.52, p < 0.01) and CF patients (r = 0.42, p < 0.05).

In children with pneumonia, sCD14 levels in BALF correlated positively with sCD14 serum levels (r = 0.32, p < 0.05), whereas in CF, asthma and control children no correlation was found. A positive correlation between sCD14 levels and WBC was found in pneumonia (r = 0.41, p < 0.05) and CF patients (r = 0.39, p < 0.05), but not in asthmatics and control subjects.

Since our results indicated that sCD14 serum levels are particularly increased in bacterial pneumonia, we analyzed the usefulness of sCD14 serum levels to differentiate bacterial pneumonia from CF, asthma and healthy controls in comparison to the traditionally used markers CRP and WBC. In this limited number of patients, soluble CD14 serum levels tended towards a higher sensitivity, specificity and diagnostic accuracy compared to CRP and WBC in the diagnosis of bacterial pneumonia (Table 2).

**Table 2:** The value of sCD14 in serum and BALF compared to CRP and WBC for diagnosing bacterial pneumonia in children.

	sCD14 serum	CRP	WBC	sCD14 BALF
Sensitivity^1 ^[%]	93.6	80.7	64.5	41.9
Specificity^2 ^[%]	100	100	96.8	96.8
Diagnostic accuracy^3 ^[%]	97.9^4^	93.6	86.0	78.5
Area under the ROC curve [%]	99.8	93.1	87.9	69.0
Cut-off value	8444 ng/ml	30 mg/l	14.0 × 10^9^	55 ng/ml

^1 ^Sensitivity is defined as the probability that a patient with pneumonia shows elevated levels of the respective marker at the specific cut-off level.

^2 ^Specificity is defined as the probability that a patient without pneumonia shows levels of the respective marker below the specific cut-off level.

^3 ^Diagnostic accuracy is defined as the number of correctly positive categorized plus the number of correctly negative categorized as percentage of all.

^4 ^The same diagnostic accuracy (97.9%) was reached for sCD 14 in serum at a cut-off value of 5429 ng/ml resulting in a sensitivity of 100% and a specificity of 96.8%.

### Two distinct cellular mechanisms facilitate sCD14 release

**Figure 6:**
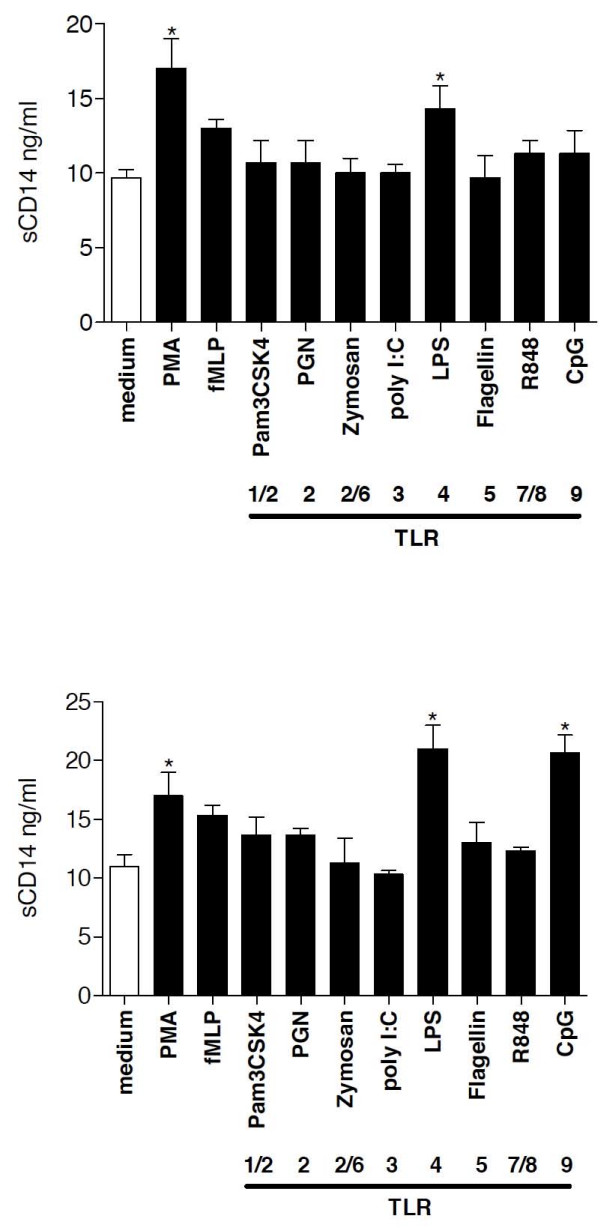
***In vitro *stimulation of PBMCs**. sCD14 levels in cell-culture supernatants were quantified in duplicates by ELISA. * p < 0.05 vs medium treatment. PBMCs (2 × 10^6^) in RPMI medium were stimulated with PMA (10 ng/ml), fMLP (100 ng/ml) or the TLR ligands CpG-DNA (100 μg/ml), zymosan (50 μg/ml), PGN (1 μg/ml), Pam_3_CSK_4 _(1 μg/ml), flagellin (1 μg/ml) or LPS (100 ng/ml) for 1 hour (A) or 40 hours (B) at 37°C.

**Figure 7:**
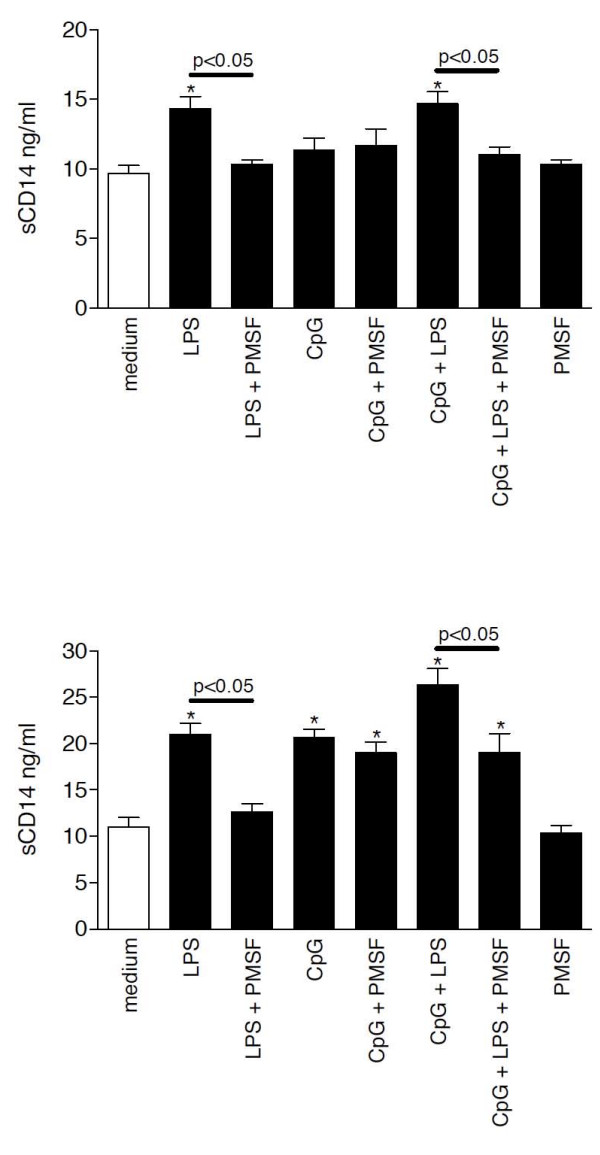
**Mechanisms of sCD14 release**. PBMCs were preincubated for 1 hour with the serine protease inhibitor PMSF (10 mM) and were then stimulated for 1 hour (C) or 40 hours (D) at 37°C with RPMI, CpG-DNA (100 μg/ml) or LPS (100 ng/ml).

**Figure 8:**
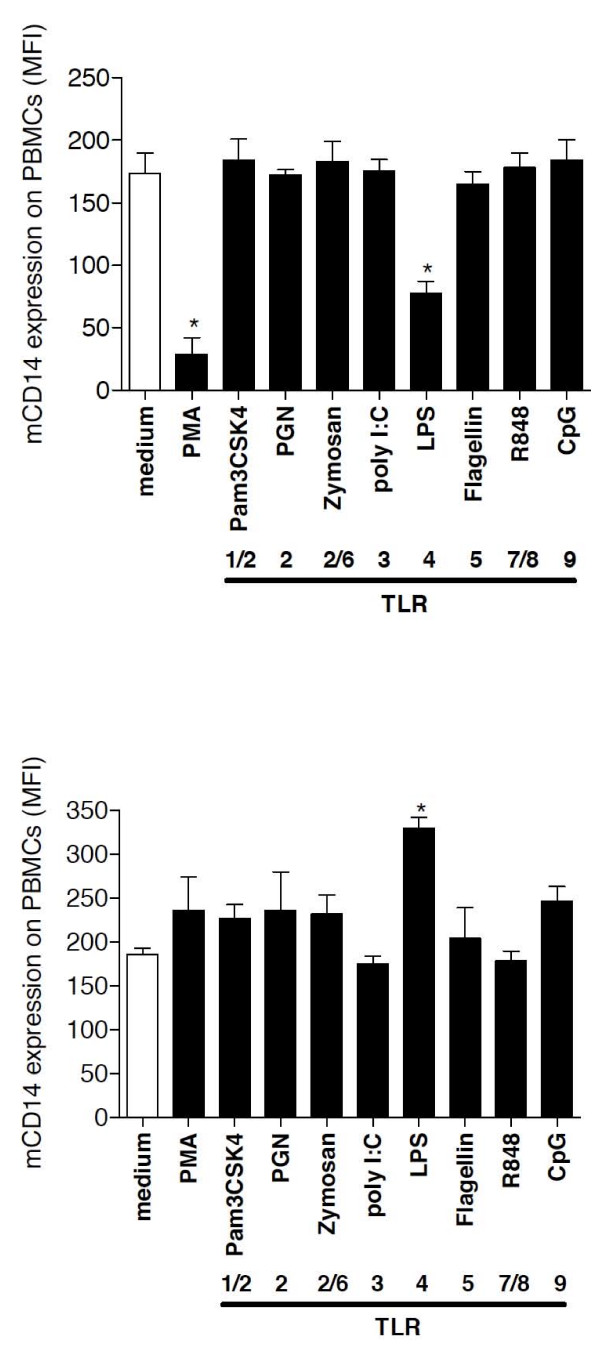
**Mechanisms of sCD14 release**. After stimulation of PBMCs (2 × 10^6^) in RPMI medium with PMA (10 ng/ml), fMLP (100 ng/ml) or the TLR ligands CpG-DNA (100 μg/ml), zymosan (50 μg/ml), PGN (1 μg/ml), Pam_3_CSK_4 _(1 μg/ml), flagellin (1 μg/ml) or LPS (100 ng/ml) for 1 hour (E) or 40 hours (F) at 37°C, mCD14 expression levels on PBMCs were quantified by flow cytometry. MFI: mean fluorescence intensity.

To recapitulate *in vitro *which factors may induce the high levels of sCD14 in serum of bacterial pneumonia patients *in vivo*, we incubated PBMCs in short-term (1 hour) and long-term culture (40 hours) conditions at 37°C with PMA, fMLP and several purified TLR ligands and analyzed sCD14 and mCD14 levels after the incubation period. After 1 hour, PMA and LPS induced sCD14 production, whereas after 40 hours of incubation, LPS and CpGs most strongly induced the release of sCD14 levels in cell-culture supernatants compared to medium treatment (Figure 6). A protease-dependent shedding and a protease-independent release of sCD14 have been described previously [10]. The mechanism of CpG-mediated sCD14 release was further investigated by pretreating the cultured cells with the serine protease inhibitor PMSF and further stimulation for 1 hour or 40 hours (Figure 7) with LPS or CpGs. Whereas LPS-mediated sCD14 release was almost completely inhibited at both incubation periods by PMSF pretreatment, the effect of CpGs on sCD14 levels after 40 hours of incubation was protease-independent since PMSF did not inhibit CpG-induced sCD14 release by PBMCs. After 40 hours of incubation, LPS and CpGs cooperated in the induction of sCD14 levels and pretreatment with PMSF partially prevented the LPS and CpG mediated sCD14 production by PBMCs. PMSF alone had no effect on sCD14 production by PBMCs. Whereas PMA and LPS decreased mCD14 expression on PBMCs after 1 hour of incubation, 40 hours of incubation with LPS increased mCD14 expression (Figure 8). No effect of CpG treatment on mCD14 expression was found after 1 hour or 40 hours of incubation.

## Discussion

This study characterizes for the first time the expression, regulation, localization and clinical significance of soluble and membrane CD14 receptors in pediatric inflammatory lung diseases. sCD14 serum levels were specifically increased in serum of children with pneumonia compared to CF, asthma and control subjects. In order to clarify which factors induce sCD14 production in bacterial pneumonia, we found that the TLR ligands LPS and CpGs induce sCD14 production via two distinct mechanisms.

Martin et al. examined sCD14 levels in BALF of adult patients with pneumonia and found increased levels of 50 ng/ml compared to ARDS and control subjects with a considerable overlap between ARDS and pneumonia patients [11,23]. Since no serum was available in these pneumonia patients, the sCD14 serum levels and their diagnostic potential remained unknown. In further studies, high sCD14 serum levels were associated with mortality in adult patients with Gram-negative [24] and Gram-positive sepsis [25]. CRP is commonly used to identify bacterial pneumonia in children and has been found to have a higher sensitivity and specificity for community-acquired pneumonia compared to WBC and ESR, especially at levels >60 mg/l [26-30]. Nevertheless, especially in children with pneumonia, the analysis of CRP has been discussed to yield unsatisfactory results [31]. In our study, limited by the small number of patients, CRP levels of >60 mg/l were present in 20 of all 32 (= 63%) children with bacterial pneumonia and had a high diagnostic accuracy of 93.6% for diagnosis of pneumonia. sCD14 levels in serum reached a diagnostic accuracy of 97.9%. This diagnostic accuracy was reached for two different cut-off levels of sCD14 in serum (5429 and 8444 ng/ml). However, increased sCD14 serum levels were not to be specific for bacterial pneumonia as sCD14 levels were also elevated in patients with non-bacterial pneumonia with a substantial overlap among patient groups. More clinical studies including higher numbers of children with pneumonia and comparing sCD14 with other putative serum biomarkers are needed to evaluate the potential role of sCD14 as biomarker for pneumonia and to define the best cut-off value for sCD14 serum levels in pediatric pneumonia.

The question arised which cells produce sCD14 and why sCD14 levels were clearly higher in serum despite pneumonia takes place primarily in the pulmonary compartment. To elucidate which factors may induce the release of sCD14 in patients with bacterial pneumonia, we stimulated PBMCs with TLR ligands *in vitro*, resembling the human situation of children with pneumonia, and quantified the resulting sCD14 production in PBMC supernatants. Among viral and bacterial TLR ligands tested, only bacterial LPS and CpG induced sCD14 production. Whereas LPS triggered sCD14 release after 1 hour, the effect of CpGs became obvious after long-term incubation. Two mechanisms of sCD14 production have been identified, a protease-dependent shedding from the cell surface and a protease-independent release of sCD14 before addition of the glycosyl phosphatidylinositol anchor [10]. Interestingly, the protease inhibitor PMSF had no effect on CpG-mediated sCD14 release. Therefore, we speculate that CpG does not induce CD14 shedding but specifically induces sCD14 release by PBMCs via a protease-independent cellular secretion mechanism as found by Bufler et al. [10]. In line with the stimulatory effect of LPS and CpG on sCD14 release by PBMCs, we speculate that sCD14 levels in patients with pneumonia might be mainly induced via the TLR4 and TLR9 ligands LPS and CpGs, respectively. The intracellular signalling pathway that triggers sCD14 elaboration upon CpG stimulation remains to be established.

We found an increased mCD14 expression on alveolar macrophages in BALF of children with pneumonia, but decreased mCD14 expression on alveolar macrophages of patients with CF compared to controls. Alexis et al demonstrated that neutrophil elastase reduced CD14 expression on alveolar phagocytes *in vitro*, which might represent the underlying cause for the low mCD14 expression on CF alveolar macrophages [32,33]. Two previous studies demonstrated that short-term incubation with LPS decreased mCD14, whereas long-term LPS treatment resulted in increased mCD14 expression on monocytes [21] and alveolar macrophages [34]. Our findings indicate that 40 h incubation with LPS and CpG induced sCD14 secretion and increased mCD14 expression of PBMCs. The LPS-induced increase of mCD14 expression might explain the high mCD14 expression found on PBMCs and alveolar macrophages from patients with pneumonia compared to CF, asthma and control subjects. In patients with arthropathies, sCD14 levels in serum correlated positively with CRP levels and sCD14 was therefore characterized as an acute-phase protein, produced mainly by hepatocytes [35]. In contrast, in children with pneumonia in our study we found no association between sCD14 levels and CRP, but instead a positive correlation with WBC, suggesting that leukocytes may represent a major source of sCD14 in serum of pneumonia patients.

Given the heterogeneous pneumonia patient cohort including patients with malignancies (especially ALL), primary immunodeficiencies (especially chronic granulomatous disease, CGD), chronic bronchitis and recurring pneumonia, the finding that sCD14 serum levels were significantly increased in the presence of pneumonia regardless of the diverse underlying pathology, suggests that sCD14 serum levels primarily reflect the pulmonary inflammation process independent of systemic disease conditions.

Nevertheless, several questions on the diagnostic value of sCD14 serum levels in childhood pneumonia remain open: (i) what is the longitudinal course of sCD14 serum levels in children with pneumonia? (ii) is there a predictive value of sCD14 levels in serum as prognostic marker for the occurrence of pneumonia in childhood? (iii) Are sCD14 serum levels increased prior to the full clinical manifestation of pneumonia or are increased sCD14 serum levels the consequence of an already existing pneumonia? These questions necessitate prospective studies in large patient cohorts.

Surprisingly, CF patients who are chronically colonized with bacterial infections had comparable sCD14 serum levels as healthy controls. We speculate that there are three possible causes for this observation: (i) sCD14 has been described as an acute phase protein. Since CF is a chronic disease, sCD14 serum levels might be exclusively increased in acute bacterial infections; (ii) elastase, present at large amounts in CF airways, has been shown to cleave mCD14 from monocytes [36] and alveolar macrophages [32]. Thus, elastase in CF lungs could decrease mCD14 and thereby reduce the mCD14 amount to be cleaved by LPS and shedded into the airway environment; (iii) the intrinsic genetic mutation in the CFTR gene might influence the expression and or release of sCD14, a hypothesis to be tested in the future.

## Conclusions

We demonstrate that sCD14 levels were increased in serum of children with pneumonia compared to CF, asthma and control subjects with the highest levels found in children with bacterial pneumonia. Functional studies revealed two distinct cellular mechanisms mediating the release of sCD14. Soluble CD14 levels may serve as a novel biomarker marker for bacterial pneumonia in children.

## Abbreviations

BALF: bronchoalveolar lavage fluid; BSA: bovine serum albumin; CF: Cystic fibrosis; CpG: nonmethylated CpG-oligonucleotides; m/sCD14: membrane/soluble CD14; ESR: erythrocyte sedimentation rate; FEV_1_: Forced expiratory volume in 1 second; LPS: Lipopolysaccharide; MFI: Mean fluorescence intensity; PBMCs: peripheral blood mononuclear cells; PGN: Peptidoglycan; PMA: Phorbol myristate acetate; TLR: Toll-like receptor; WBC: white blood cell count.

## Competing interests

The authors declare that they have no competing interests.

## Authors' contributions

VM carried out the experimental analyses and wrote the manuscript. PL performed statistics. MG and AH characterized the study population, performed bronchoalveolar lavage and participated in the study design. TN performed bronchoalveolar lavage and patient characterization. S.S. M.L., F.H. B.K. and P.B. contributed to the *in vitro *studies and analyzed data. DH and MG designed the study, supervised the experimental analyses and wrote the manuscript. All authors read and approved the final manuscript.
